# False positives in neuroimaging genetics using voxel-based morphometry data

**DOI:** 10.1016/j.neuroimage.2010.08.049

**Published:** 2011-01-15

**Authors:** Matt Silver, Giovanni Montana, Thomas E. Nichols, the Alzheimer's Disease Neuroimaging Initiative

**Affiliations:** a Statistics Section, Department of Mathematics, Imperial College London, UK; b Department of Statistics & Warwick Manufacturing Group, University of Warwick, UK

## Abstract

Voxel-wise statistical inference is commonly used to identify significant experimental effects or group differences in both functional and structural studies of the living brain. Tests based on the size of spatially extended clusters of contiguous suprathreshold voxels are also widely used due to their typically increased statistical power. In “imaging genetics”, such tests are used to identify regions of the brain that are associated with genetic variation. However, concerns have been raised about the adequate control of rejection rates in studies of this type. A previous study tested the effect of a set of ‘null’ SNPs on brain structure and function, and found that false positive rates were well-controlled. However, no similar analysis of false positive rates in an imaging genetic study using cluster size inference has yet been undertaken.

We measured false positive rates in an investigation of the effect of 700 pre-selected null SNPs on grey matter volume using voxel-based morphometry (VBM). As VBM data exhibit spatially-varying smoothness, we used both non-stationary and stationary cluster size tests in our analysis. Image and genotype data on 181 subjects with mild cognitive impairment were obtained from the Alzheimer's Disease Neuroimaging Initiative (ADNI). At a nominal significance level of 5%, false positive rates were found to be well-controlled (3.9–5.6%), using a relatively high cluster-forming threshold, *α*_*c*_ = 0.001, on images smoothed with a 12 mm Gaussian kernel. Tests were however anticonservative at lower cluster-forming thresholds (*α*_*c*_ = 0.01, 0.05), and for images smoothed using a 6 mm Gaussian kernel. Here false positive rates ranged from 9.8 to 67.6%. In a further analysis, false positive rates using simulated data were observed to be well-controlled across a wide range of conditions.

While motivated by imaging genetics, our findings apply to any VBM study, and suggest that parametric cluster size inference should only be used with high cluster-forming thresholds and smoothness. We would advocate the use of nonparametric methods in other cases.

## Research Highlight

►RFT nonstationary cluster size test on VBM is found to be invalid based on empirical null studies. ►Invalid inferences (inflated false positive risk) were found with 3 voxel smoothing. ►With 6 voxel smoothing, invalid inferences were found with 6 voxel smoothing and alpha=0.01 cluster-forming threshold. ►RFT nonstationary test only found to be valid with 6 voxel smoothing with alpha=0.001 cluster-forming threshold. ►Equivalent Gaussian data simulations produced valid inferences, suggesting VBM data violates RFT assumptions and motivates the use of nonparametric permutation methods.

## Introduction

In imaging genetics brain images are used as phenotypes that are modelled by genetic predictors. In contrast to conventional genetic association studies where disease status is based on clinical observations, neuroimaging phenotypes are able to capture aspects of disease phenotypes at the physiological level. Neuroimaging genetic studies thus offer the prospect of gains in statistical power, since genes code not for mental or behavioural traits, but for the neural phenotypes that underpin them (Glahn et al., 2007). Neuroimaging genetic studies have the additional benefit of spatially localising gene effects, offering further potential insights into the structural and functional neurobiology of disease (Meyer-Lindenberg and Weinberger, 2006; Roffman et al., 2006; Hariri et al., 2006). Structural and functional brain images have been used in candidate and whole genome investigations of a number of neurological disorders including schizophrenia (Roffman et al., 2006; Potkin et al., 2009a, b) and Alzheimer's Disease (Filippini et al., 2009; Pievani et al., 2009), and a recent study has highlighted gene effects on cortical surface area (Joyner et al., 2009).

A range of statistical methods have been used to investigate associations between SNPs and structural and functional neuroimages. Techniques include linear regression, parallel independent component analysis and multivariate approaches that model the influence of multiple SNPs at a time (Ashburner and Friston, 2000; Calhoun et al., 2009; Hardoon et al., 2009). The problem of multiple testing presents a particular challenge in imaging genetic studies, with a whole-brain study of 700 SNPs across 200,000 voxels requiring 1.4 million separate tests, raising concerns about false positives. Random field theory (RFT) is a widely used multiple testing method for determining corrected significances while accounting for spatial dependencies between voxels (Worsley et al., 1996a). These correlations are introduced by the image acquisition process itself, by physiological signal not included in the model, by image resampling during re-alignment, or by explicit smoothing applied in pre-processing (Frackowiak et al., 2003, chap. 14). Such RFT techniques must however be tested on representative empirical data before their efficacy can be firmly established. In a recent study Meyer-Lindenberg et al. (2008) measured rejection (i.e. false positive) rates in an imaging genetic study using voxel-wise inference against a pre-selected set of ‘null’ SNPs considered to have no effect on brain structure or function. Subjects included patients with schizophrenia, as well as healthy controls. Gene effects on brain structure (using VBM) and function (using fMRI response to cognitive tests) were considered. The study looked at false positive rates both across the brain as a whole, and for specific regions of interest. Significance thresholds were adjusted for comparisons across multiple voxels using both family-wise error (FWE) and false discovery rate (FDR) corrections. At a nominal significance level of 0.05, they found empirical rejection rates ranging from 0.2 to 4.1%, suggesting that for the methods studied, false positive rates are well-controlled, and that inferences are if anything conservative.

### Cluster size tests

A variety of approaches are used to identify significant signals in the brain. In voxel-wise tests, group differences or differences in activation are assessed at each individual voxel, so that locations where there is a strong association between voxel intensity and a disease phenotype for example, are labelled as significant. In cluster size tests, an arbitrary cluster-forming threshold is applied to define connected components, and then significance is assessed on the basis of the size of the clusters. Cluster size tests are relatively more sensitive than voxel-wise tests for spatially extended signals (Friston et al., 1996; Poline et al., 1997; Moorhead et al., 2005), since they make use of the spatial nature of the signal and require a less severe multiple testing correction (there are always fewer clusters than voxels). This necessarily comes at the cost of reduced localising power, as rejecting the cluster null hypothesis just implies that one or more voxels within the cluster are significant.

As with voxel-wise tests, cluster size tests must account for the fact that neighbouring voxels are correlated even without any true effects RFT is used to assign *P*-values to each cluster in the statistic image accounting for smoothness and search volume. However, RFT rests on a number of assumptions, and simulation studies have shown that the performance of this technique depends on the choice of cluster-forming threshold, and on the use of sufficiently smooth images (Hayasaka and Nichols, 2003).

Inferences using cluster size are also subject to confounding effects arising from ‘non-stationarity’ — i.e., from local variations in noise smoothness. Under non-stationarity, even when there is no signal present, clusters will be larger in ‘smoother’ regions, and smaller in ‘rougher’ ones. Methods that fail to take such local non-stationarity into account will provide unreliable inferences, with areas of extended smoothness producing large clusters and increased incidence of false positives, and conversely rough areas demonstrating greater incidence of false negatives (Hayasaka et al., 2004). One approach to tackling this problem is to adjust cluster sizes according to local smoothness using non-stationary RFT methods (Worsley, 2002).

Hayasaka et al. (2004) compared stationary and non-stationary RFT cluster size inference methods in the identification of activated areas using simulated and PET data sets. They found that the stationary RFT method was anticonservative^2^ under non-stationarity, but that the non-stationary RFT test performed well only for smooth images under high degrees of freedom. While that work suggested the use of corresponding nonparametric non-stationary cluster size permutation tests, here we are motivated to use parametric RFT in the imaging genetics context, as testing massive numbers of SNPs may make permutation tests impractical.

In this study we measured false positive (type I error) rates for voxel-wise and cluster size neuroimaging genetic inference on a dataset comprising 181 MRI images and associated genotype information from the Alzheimer's Disease Neuroimaging Initiative (ADNI) online database. We follow a similar experimental design to that of Meyer-Lindenberg et al. (2008), although we restrict our analysis to genotypic effects on whole-brain structure (using VBM). We supplement our real data with simulated data evaluations to aid in the interpretation of the real VBM data results.

## Methods and materials

Real imaging and genotype data were obtained from the Alzheimer's Disease Neuroimaging Initiative (ADNI) database (www.loni.ucla.edu/ADNI). The ADNI was launched in 2003 by the National Institute on Aging (NIA), the National Institute of Biomedical Imaging and Bioengineering (NIBIB), the Food and Drug Administration (FDA), private pharmaceutical companies and non-profit organizations. ADNI is the result of efforts of many co-investigators from a broad range of academic institutions and private corporations, and subjects have been recruited from over 50 sites across the U.S. and Canada (see Acknowledgments for more details).

### Imaging data

#### ADNI subjects

181 T1-weighted 3D structural MRI scans from subjects with mild cognitive impairment (MCI) were obtained from the ADNI database (see Jack et al. (2008) for image acquisition details). In the present study, SPM5's (http://www.fil.ion.ucl.ac.uk/spm/software/spm5) unified segmentation and normalisation was used to obtain gray matter (GM) images in standard space, modulated to account for volume changes in the warping to the MNI atlas. Modulated GM images were smoothed with 6 mm and 12 mm Gaussian kernels. 12 mm smoothing is the de-facto standard in VBM studies (Ashburner and Friston, 2000), and was used by Meyer-Lindenberg et al. (2008). A second set of images with 6 mm smoothing enables the performance of RFT at relatively low smoothness to be assessed. A grey matter analysis mask was constructed by thresholding the mean grey matter image at 0.025. All pre-processing and smoothing was carried out using SPM5.

#### Simulated images

Stationary and non-stationary random images were generated using FSL (http://www.fmrib.ox.ac.uk/fsl/). 3D simulated images had the same voxel size (2mm^3^) as MNI-warped ADNI images, and were also masked with the real data analysis mask.

**Fig. 1:**
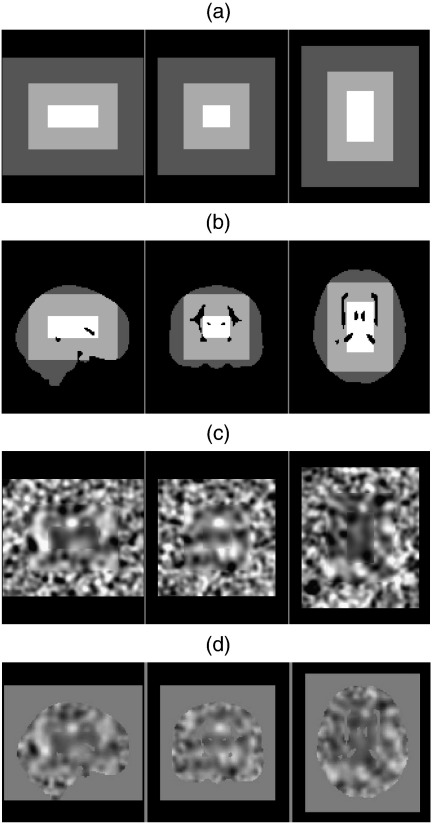
Non-stationary image simulation. (a) Schematic illustrating extent of 3 different smoothness regions. (b) as (a) with ADNI image brain mask applied. (c) Realisation of non-stationary image with outer, middle and inner regions smoothed with 8, 12 and 18 mm FWHM Gaussian smoothing kernels. (d) as (c) with final 1.5 mm smoothing kernel and ADNI mask applied.

Stationary realisations were generated using white noise images convolved with 6 mm and 12 mm 3D Gaussian smoothing kernels. Non-stationary realisations were generated from white noise images smoothed with 3 different Gaussian kernels extending over distinct, adjacent 3D regions of the image volume. “6 mm” non-stationary images were composed of a central region smoothed with a 9 mm kernel, with intermediate and outer regions smoothed with 6 and 4 mm kernels respectively. “12 mm” images were made up of regions smoothed with 12, 8, and 18 mm kernels (see Fig. 1). Final images were smoothed with a 1.5 mm kernel to eliminate discontinuities at the boundaries between different regions, resulting in final non-stationary smoothness of 4.3, 6.2 and 9.1 mm FWHM for “6 mm”, and 8.1, 12.1 and 18.1 for “12 mm”. All images were truncated from a larger initial volume to avoid edge artefacts. Finally, to match the real data under consideration, only voxels within the real-image grey matter mask were used.

### Genotype data

In their study of genetic effects on brain structure, Meyer-Lindenberg et al. (2008) selected 720 ‘null’ SNPs, found to have no significant association with disease phenotype (at the 5% level) in separate case–control and family-based analyses. The results of the subsequent neuroimaging genetic analysis were considered to set an upper bound on null rejection rates, since individual SNPs may still influence brain structure after all. To establish a lower bound, the authors repeated their analysis, but with the genotype–phenotype relationship removed by permuting genotypes across subjects. 4 such permutations were analysed. In the present study, 700 selected ‘null’ SNPs are used, with 10 subsequent permutations.

ADNI subjects' genotype information, assayed using the Illumina 610-Quad BeadChip microarray, was obtained from the ADNI website. Each genotype file contains information pertaining to 620,901 SNPs and copy number variations (CNVs). 700 ‘null’ SNPs were selected as follows. Firstly, all CNVs were excluded and only SNPs from chromosome 3 were considered. Chromosome 3 was chosen since none of 4 prime candidate AD-associated genes (APOE, PSEN1, PSEN2 and SORL1) are located on this chromosome. Of the remaining 39,928 SNPs, those with a minor allele frequency of less than 5% were excluded, as were any SNPs with a Bonferroni-corrected Hardy Weinberg equilibrium *P*-value < 0.05/700. From the remaining 18,285 SNPs, 700 uniformly-spaced (by rank order in position) SNPs were selected, in order to minimise any possible linkage disequilibrium effects. Finally, as per standard practice, an adjustment was made to those SNPs (310 in total) with low numbers (< 10) of homozygous alleles, merging the rare homozygous and heterozygous groups. This is to minimise any potential biasing effects in the regression, and is equivalent to fitting a dominant or recessive model at the SNPs in question.

### Statistical inference

Voxel-wise and cluster size tests for association between genotype and grey matter intensity were performed under the General Linear Model (GLM) (Friston et al., 1995) using SPM5. Non-stationary tests were carried out using Hayasaka's non-stationary toolbox for SPM (http://fmri.wfubmc.edu/cms/NS-General). The non-stationary toolbox corrects for expected variation in cluster size in non-stationary images under the null, using statistical random field theory (Hayasaka et al., 2004; Worsley et al., 1999). Note that we did not compare our results with standard permutation tests (Hayasaka et al., 2004). Permutation methods are guaranteed to be valid under the null hypothesis, and our prime motivation in this study was to address the accuracy of RFT methods in a large data (i.e. imaging genetics) setting where permutation might not be practical computationally.

For the real (ADNI) image dataset, genotype effects were measured by modelling modulated grey matter intensity as a response to SNP allele frequency, with subject age and sex as nuisance covariates. Each SNP was analysed separately, with SNP significance determined from *t* and *F*-tests, corrected for multiple comparisons using family-wise error and false discovery rate. For cluster size tests, cluster-forming thresholds, *α*_*c*_, of 0.001, 0.01 and 0.05 under both stationary and non-stationary assumptions were considered. Overall rejection rates express the proportion of the 700 SNPs found to cause any significant activation. All tests were repeated a total of 10 times with genotype–phenotype labels permuted to remove any possible remaining association.

Equivalent tests on simulated images were conducted with the same SNP and covariate (age, sex) data, so that degrees of freedom for all tests were the same as those on the ADNI dataset. These tests were performed without permutation since there can be no association between genotype and phenotype with random images.

#### Non-stationary cluster size inference

While the RFT non-stationary cluster size test is described in detail elsewhere (Hayasaka et al., 2004), we review it again here to facilitate later discussion. Under the GLM, the intensity *Y(v)* at voxel location *v* is expressed as a linear combination of regressors

(1) Y(v)=Xβ(v)+ε(v)

where, in a study with *n* subjects and *p* regressors, *X* is an *n* ×  *p* design matrix, *β*(*v*) is a *p*-dimensional vector of parameters to be estimated, and *ε*(*v*) is an *n*-vector of error terms, assumed to be independent and normally distributed with equal variance.

With cluster size tests, significant clusters are formed from contiguous voxels whose *t* or *F*-statistic exceed a fixed cluster-forming threshold, *u*_*c*_ (or equivalently, an uncorrected significance level *α*_*c*_ that uniquely determines *u*_*c*_). Briefly, the non-stationary toolbox corrects for image non-stationarity by measuring the ‘smoothness’ at each voxel, a quantity that is related to the variance of the spatial partial derivatives of the model errors, *ε* in (1). From this a measure of image smoothness, measured in FWHM is obtained. FWHM refers to the ‘full-width at half-maximum’ of a Gaussian kernel required to smooth a random (white noise) image into equivalent smoothness of the data at hand; note that isotropy is not assumed, and FWHM is fully specified by [FWHM_*x*_ FWHM_*y*_ FWHM_*z*_]. A related quantity is the RESEL, a ‘virtual’ voxel of size FWHM_*x*_ × FHWM_*y*_ × FHWM_*z*_. The RESEL count *N*_*res*_ is the number of RESELs that fit into the search volume,

(2) $$N_{\mathrm{res}}=\frac{V}{{\text{FWHM}}_{x}\times {\text{FHWM}}_{y}\times {\text{FHWM}}_{z}}$$

where *V* is the number of voxels in the image. When stationarity (i.e. uniform smoothness across the image) is assumed, FWHM is calculated by pooling FWHM across the entire image volume.^3^ Under non-stationarity, FWHM is estimated at each voxel *v*, giving a RESEL measure as well. The size of this local RESEL, 1/ [FWHM_*x*_(*v*) × FHWM_*y*_(*v*) × FHWM_*z*_(*v*)], is denoted RPV *(v)* for RESELs per voxel at voxel *v*. In this way a voxel's effective volume, relative to image smoothness, is obtained. The next step is to calculate the smoothness-adjusted cluster size, *S*^′^, by summing effective voxel volumes over a cluster:

$$S^{\text{′}}=\sum_{v\in C}\;\text{RPV}(v)$$

where *C* denotes the set of voxel indices in the cluster. This procedure is equivalent to measuring cluster size in a distorted image, where space has been warped in such a way so as to ensure that stationarity holds (Worsley et al., 1999).

Finally, the probability of obtaining clusters of a given size *S*^′^ under the null is calculated, corrected for multiple comparisons. This probability is derived from the image's Euler Characteristic, *ρ*(*u*_*c*_), a topological property which approximates the expected number of clusters or ‘blobs’ in a thresholded image of given smoothness. In the stationary case, the expected cluster size under the null is

(3) $$E(S)=\frac{E(N_{v})}{E(C)}$$

where $E(N_{v})$ is the expected number of suprathreshold voxels (= *Vα*_*c*_), and E(C) is the expected number of clusters (= *Vρ*(*α*_*c*_)). This expression also holds for *S*^′^ but suprathreshold voxels must be measured in RESELs, i.e. $E(N_{v})=N_{\mathrm{res}}\alpha_{c}$. The expected cluster size is then used to estimate the null distribution of *S* (or *S*^′^) and obtain uncorrected *P*-values, which are then converted to either FWE-corrected *P*-values or FDR-corrected *P*-values (Chumbley and Friston, 2009) that account for searching the brain for significant clusters.

The use of RFT in cluster size tests rests on a number of assumptions (Hayasaka and Nichols, 2003). These include:

• Lattice approximation — images are assumed to be derived from a smooth random field sampled at regular points on a lattice; sampling is assumed to be fine enough to capture the local features of the field;
• Image smoothness — images are smooth at the voxel scale;
• Large search region — Search volume is large compared to the size of a resel;
• Uniform smoothness (for stationary tests only);
• High cluster-forming thresholds — RFT's estimate of cluster size distribution under the null is derived asymptotically, under the assumption that the cluster-forming threshold *α*_*c*_ is sufficiently high.

These assumptions present particular practical difficulties for those using cluster size tests, since low thresholds with as little smoothing as possible — the very conditions under which RFT performs worst — tend to maximise sensitivity and localising power (Hayasaka and Nichols, 2003).

## Results

### Real (ADNI) images

Full cluster and voxel-wise results under FWER correction are presented in Table 1. Similar results under FDR correction are presented in Table 2. Relevant whole brain, voxel-wise rejection rates reported by Meyer-Lindenberg et al. (2008) are also included for comparison.

Results from tests with permuted genotype–phenotype labels (FWER-corrected results only) were broadly similar to those with observed, unpermuted labels, indicating that for the purposes of the present study, chromosome 3 SNP effects on brain structure were negligible.

The key finding was that rejection rates were poorly controlled for all cluster size tests, except for those performed on 12 mm smoothed images with the highest cluster-forming threshold, *α*_*c*_ = 0.001. In this latter instance, FWER-corrected rejection rates approached the desired nominal 5% level, with a 3.8 ± 0.8% rejection rate for a *t*-test with non-stationary correction under permutation, and 4.5 ± 1.2% under the corresponding *F*-test. FDR-corrected results were broadly similar to FWE-corrected results for *α*_*c*_ = 0.01 and 0.001.

FWE and FDR-corrected voxel-wise tests were conservative for both 6 and 12 mm smoothed images, in agreement with results for FWER-corrected voxel-wise *t*-tests on 12 mm smoothed images reported by Meyer-Lindenberg et al. (2008).

In general, cluster size tests became more anticonservative at lower thresholds (decreasing *u*_*c*_, increasing *α*_*c*_), and this effect was exacerbated for low smoothness images. Image smoothness had a pronounced effect on all results, with tests performed on 6 mm smoothed images having substantially higher rejection rates than those performed on images with 12 mm smoothing. The degree of smoothing, however, showed little effect on voxel-wise rejection rates.

Cluster size tests corrected for image non-stationarity were generally closer to nominal than those assuming stationarity. Finally, *F*-tests were generally more anticonservative than equivalent *t*-tests.

### Simulated images

Rejection rates for tests on simulated, random Gaussian images are presented in Table 3. For stationary (constant smoothness) 6 and 12 mm FWHM Gaussian images, both stationary and non-stationary cluster size *t*-tests are highly conservative at higher thresholds (*α*_*c*_ = 0.001, 0.01), but are anticonservative at the lowest threshold (*α*_*c*_ = 0.05). *F*-tests are conservative at all thresholds. As with stationary images, non-stationary cluster size *t*-tests are conservative at *α*_*c*_ = 0.001, 0.01, and anticonservative at *α*_*c*_ = 0.05, whereas *F*-tests are conservative at all thresholds. As might be expected, stationary cluster size *t* and *F*-tests on both “6 mm” and “12 mm” FWHM non-stationary images perform poorly.

Voxel-wise tests are generally conservative, or close to nominal for both stationary and non-stationary images at 6 and 12 mm.

## Discussion

This study provides the first analysis of false positive rates in an imaging genetics study of VBM data using cluster size inference. Images from a group of 181 subjects with mild cognitive impairment were tested against a set of 700 ‘null’ SNPs. The analysis presented here suggests that rejection rates under both stationary and non-stationary assumptions are poorly controlled at low cluster-forming thresholds or for images with low smoothness.

The use of real genotype data is considered important, since the accurate modelling of linkage disequilibrium and population stratification is a challenge, and their effect on neural phenotypes is unknown (Meyer-Lindenberg et al., 2008).

Null SNPs were selected from chromosome 3, with the simple rationale that none of the genes reported to have the strongest link with AD are present on this chromosome. While this is clearly a crude measure for selecting SNPs with no effect on grey matter distribution, the use of multiple permutations ensures that any possible effects are removed by breaking the association between genotype and phenotype. In fact, rejection rates obtained using permuted SNPs are not significantly different from those obtained without permutation (considering a 95% confidence interval at ± 2 SD), indicating that, for the purposes of this study, SNP effects on brain structure are indeed negligible.

We begin by considering the results obtained with the ADNI image dataset.

### Effect of cluster-forming threshold, *α*_*c*_

The choice of cluster-forming threshold, *α*_*c*_ was found to have a significant effect on cluster size inference rejection rates. For images smoothed with a 12 mm Gaussian kernel, both stationary and non-stationary tests were found to be well-controlled or conservative at the most stringent threshold (*α*_*c*_ = 0.001). However, tests became increasingly anticonservative at lower thresholds *u*_*c*_ (higher *α*_*c*_) for both 12 mm and 6 mm smoothed images.

**Fig. 2:**
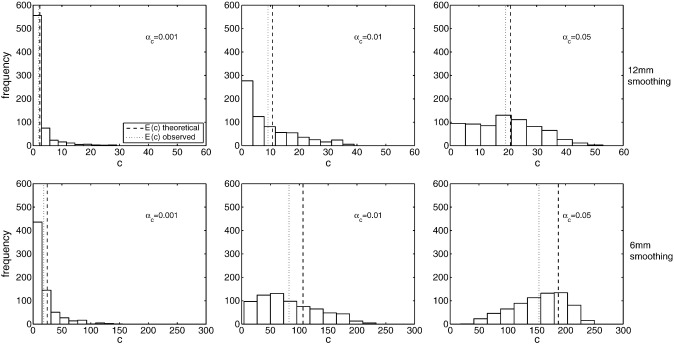
Accuracy of estimation of *c*, the theoretical number of clusters under RFT. Histograms show the empirical distribution of *c* across all 700 SNPs at three different cluster-forming thresholds (left to right), and with two different smoothing kernels (top and bottom). The theoretical (RFT) and empirical mean number of clusters, E(C), are shown as dashed and dotted lines respectively. The amount by which RFT overestimates E(C) increases as the cluster-forming threshold *u*_*c*_ is lowered, and with images of lower smoothness. (Note that the x axis for 6 mm smoothed images has a larger range, reflecting the fact that many more clusters are observed).

A possible explanation for the poor performance at low *u*_*c*_ is bias in RFT's estimate of the expected number of clusters, E(C) (Fig. 2). If E(C) is over-estimated, expected cluster size is under-estimated (see Eq. (3)), meaning that more clusters of a given size are labelled as significant. This over-estimation of E(C) may reflect the inability of the Euler Characteristic, *ρ*(*u*_*c*_), to accurately estimate the number of clusters at low thresholds, where clusters are more numerous and tend to coalesce to form topologically complex patterns (Taylor and Worsley, 2008).

### 12mm vs. 6 mm smoothing kernels

The application of a wide range of Gaussian smoothing kernels in VBM is evident in the literature — e.g. 4 mm (Schwartz et al., 2010), 8 mm (Folley et al., 2010) and 10 mm (Shen et al., 2010), as well as the ‘standard’ 12 mm (Rosen et al., 2010; Ueda et al., 2010). However guidelines on the particular choice of smoothing kernel have been described as ‘vague’ (Hayasaka and Nichols, 2003), and there is a suggestion that kernel widths should be determined empirically (Worsley et al., 1996b). Notably, with the use of high-dimensional warping methods like DARTEL (Ashburner, 2007), there appears to be a trend towards lower smoothing kernels. Improved intersubject alignment means there is a reduced need for smoothing to ‘blur out’ warping errors. For example, Bergouignan et al. (2009) use 12 mm smoothing with SPM's standard normalisation and 8 mm with DARTEL. While reduced smoothing should increase sensitivity to effects of smaller size by “Matched Filter” arguments, cluster size tests are most sensitive to effects that are larger than the noise smoothness. Hence, to the extent that large scale anatomical effects are present after either low- or high-resolution warping, high-resolution results may be more sensitive as effects will be larger in units of resels.

In the present study, differing amounts of smoothing were found to have a pronounced effect on rejection rates. Tests on images smoothed with a 6 mm Gaussian kernel were highly anticonservative at all thresholds including the highest (*α*_*c*_ = 0.001), and were consistently more anticonservative when compared with 12 mm smoothing results.

Poor performance for low smoothness images is in fact to be expected under the lattice assumption of random field theory (Hayasaka and Nichols, 2003). As image smoothness decreases, this lattice approximation breaks down, since the underlying variation is poorly-captured by discrete, voxel-wise sampling. This means that continuous RFT results are modelling unobserved, large intensity changes between sampled voxels. While previous reports have suggested 3 voxel FWHM smoothing (i.e. 6 mm FWHM smoothing for the 2 mmvoxels considered here) is sufficient (Nichols and Hayasaka, 2003), for the ADNI data this is insufficient. Specifically, we find an over-estimation of the expected number of clusters, with the gap between expected and observed values, E(C)−C, generally greater at 6 mm than at 12 mm (see Fig. 2).

### Stationary vs. non-stationary tests

**Fig. 3:**
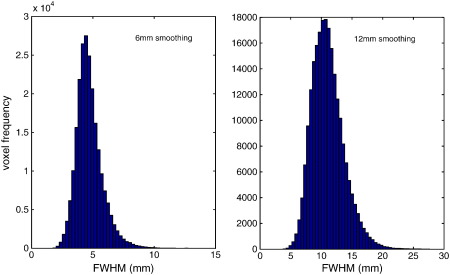
Distribution of voxel-wise FWHM for ADNI images smoothed with 6 mm (left) and 12 mm (right) Gaussian smoothing kernels. Voxel-wise FWHM gives an indication of local smoothness and corresponds to the ‘full-width at half-maximum’ of a Gaussian kernel required to produce a random (white noise) image of equivalent smoothness. A perfectly stationary image would have constant FWHM at all voxels. In contrast, a highly non-stationary image would have a large spread in FWHM, as is seen here.

Non-stationary cluster-wise rejection rates were generally similar, or slightly better-controlled than those assuming stationarity, suggesting that there is at least some non-stationarity present in the images. For non-stationary images, stationary tests would also be expected to perform worse at lower thresholds where clusters are larger and more likely to encompass extra-smooth regions, and this is indeed the case. A heuristic measure of image non-stationarity was obtained by plotting the distribution of voxel-wise FWHM, obtained from the RPV image produced by SPM (FWHM = RPV^ − 1/3^). A completely stationary image would be expected to have constant FWHM across the entire image volume. Any pronounced departure from this suggests non-stationarity. An analysis of 6 mm and 12 mm FWHM images (see Fig. 3) finds a spread of around 4 mm to 8 mm and SD of 1.0 mm for 6 mm smoothed images, and 7 mm to 17 mm and SD of 2.6 mm for 12 mm images. While this spread of FWHM could be attributed to sampling variation, the theoretical SD of the FWHM estimator can be computed by simulation (see Appendix B of Hayasaka et al. (2004)). We find theoretical SDs of 0.696 mm for 12 mm smoothed stationary images, and 0.348 mm for 6 mm images, which are much smaller than our observed values. While these theoretical SDs under stationarity again depend on the accuracy of the RFT results (and, note in particular the bias in the smoothness estimation for 6 mm smoothing), they provide further evidence of substantial image non-stationarity.

### *t* vs. *F* image results

The *t* and *F* image cluster size results cannot be directly compared. While the single degree-of-freedom *F*-test we used is exactly equal to the square of the *t*-test used, the set of clusters generated will be different for two reasons. First, the one-sided *α* level used to define a *t* statistic threshold will not equal the square root of the *F* statistic threshold of the same *α* level (an *F*'s level corresponds to the *t*'s two-sided *α* level). Further, the *F* image has the clusters arising from negative *t* values. Thus there will be both more and different clusters in the *F* images for the same data and *α*_*c*_.

These caveats aside, the rejection rates on the real data were largely similar for the same *α*_*c*_'s, with valid performance found only for 12 mm smoothed data with *α*_*c*_ = 0.001.

### Simulated images

In marked contrast to tests performed on the ADNI image dataset, non-stationary cluster size tests on simulated stationary and non-stationary random images were found to be valid (conservative) at both high and moderate cluster-forming thresholds (*α*_*c*_ = 0.001, 0.01), irrespective of image smoothness.

Other studies using simulated images produced from stationary and non-stationary, Gaussian random fields have also considered the effect of varying both the cluster-forming threshold and image smoothing kernel. With stationary simulated images, Hayasaka and Nichols (2003, Fig. 2) found that cluster size tests were conservative over the same range of image smoothness with *α*_*c*_ = 0.001, 0.01, in agreement with our results. Using similar non-stationary simulated data, Hayasaka et al. (2004) also found that non-stationary cluster size tests were conservative with images of low smoothness (comparable to our 6 mm non-stationary images), and with 20 subjects, but only considered *α*_*c*_ = 0.01.

The large discrepancy in cluster size inference rejection rates between real and simulated image data over a range of thresholds and smoothing kernels suggests that there are features of the real VBM data that may be incompatible with the RFT method. This may for example be due to the inherent non-normality of VBM data, or to patterns of non-stationarity in real images that are more complex than those simulated here. Non-normality of VBM data has been reported before, but only when considering the accuracy of voxel-wise significance (Viviani et al., 2007; Salmond et al., 2002). This other work found that imbalanced group comparisons required 12 mm FWHM smoothing to accurately control voxel-wise false positives, though balanced group comparisons were accurate with smaller kernel sizes. As genotypes are rarely equally frequent, the imbalanced results are most relevant to this setting.

We performed a number of additional simulations in order to investigate the role of non-normality in cluster size inference. VBM data is hard bounded between 0 and 1, and modulated VBM nearly so. A Shapiro–Wilks test for normality at each voxel, using the spmd5beta diagnostic toolbox (http://www.sph.umich.edu/~nichols/SPMd/) reveals that both 6 mm and 12 mm smoothed images are indeed highly non-normal. This deviation is particularly marked for 6 mm images, with around 45% of voxels exceeding a nominal 5% Shapiro–Wilks threshold. In contrast, the stationary Gaussian noise-derived simulated images describe earlier show no significant deviation from normality. To test the effect of introducing non-normality to our simulations, we generated a set of images by first thresholding Gaussian noise images smoothed with 6 mm and 8 mm kernels, to produce ‘patchy’, binary images. These were then smoothed with 6 mm and 12 mm kernels to produce images with a range of deviations from non-normality that mimicked or exceeded the deviations from normality exhibited by the real VBM data, as measured with a Shapiro–Wilks test. Regression of these images against all 700 SNPs produced similar results to those described earlier, with conservative results at high and moderate cluster-forming thresholds with both 6 mm and 12 mm smoothing.

To test the effect of more complex patterns of non-stationarity, we segmented FWHM images derived from 6 mm and 12 mm smoothed ADNI images to produce a set of topologically complex masks corresponding to regions of high, medium and low ‘smoothness’. Non-stationary simulated images were then generated by filling each masked region with differently smoothed Gaussian noise, as described in the Section Imaging data. Once again, a full analysis produced conservative results, with rejection rates below a nominal 5% for *α*_*c*_ = 0.001, 0.01 for both 6 mm and 12 mm smoothed images.

One final set of simulated images was produced by again generating complex, non-stationary FWHM-segmented masks, this time filled with non-normal, Gaussian noise-derived data, as described earlier. Rejection rates were again well-controlled, in marked contrast to results obtained using real ADNI data.

A reviewer raised the concern that poor performance might be attributable to the low (2.5%) threshold applied to the mean GM image to create an analysis mask. To address this we ran an additional set of tests using a mask based on a 20% GM threshold. This higher threshold will exclude voxels with the least amount of GM and those likely to have non-Gaussian errors, but also will change the topology of the search region, making it more convoluted. While we did find a slight improvement in test performance with the new mask on real ADNI data, our findings were left unchanged, in that only tests performed on 12 mm smoothed images with *α*_*c*_ = 0.001 were well-controlled.

## Conclusion

We found that RFT non-stationary cluster size tests on real VBM data perform poorly at low cluster-forming thresholds and for images with low smoothness. In a second analysis with synthetic image data generated using Monte Carlo simulations, we found performance was instead excellent, if conservative. The contradictory results indicate there are features of the real VBM data that are incompatible with the RFT method.

**Fig. 4:**
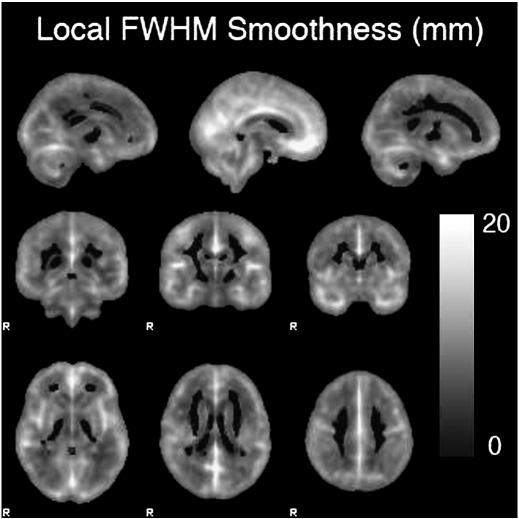
12 mm VBM image non-stationarity. The figure illustrates the variation in image smoothness measured in FWHM, derived from the SPM RPV image. There is a wide variation, ranging from 3.8 to 27.7 mm.

We suggest two possible reasons for this difference in performance. First, as grey matter segmented data is hard bounded between 0 and 1, and modulated VBM data nearly so, the data may exhibit non-normality, violating a foundational assumption of the RFT method. Second, while we simulated non-stationarity, the pattern of non-stationarity observed in real VBM is substantially more complex (Fig. 4). However, further tests using simulated images with both significant deviations from normality, and with more complex patterns of non-stationarity still produced conservative results, so that we were unable to find evidence that either aspect of real VBM data is responsible for the poor performance observed with real image data.

There are many ways to characterise deviations from normality in image data, and it may be that the VBM data deviates from normality in ways which we have been unable to capture in our simulations. The same is true of our attempts to model the true complexity of non-stationarity. Additionally, while RFT assumes that images can be warped to approximate stationarity, for VBM these hypothetical warps could be so convoluted so as to render the constituent approximations inaccurate.

Fortunately, an alternative to parameteric, RFT-based cluster size inference is available — a nonparametric permutation test where the data itself is used to derive an empirical cluster size distribution under the null (Hayasaka et al., 2004). While this approach carries a greater computational burden, the false positive rates are exact (Hayasaka and Nichols, 2003), and the permutation approach should be reasonable for studies examining only a small number of SNPs.

## Figures and Tables

**Table 1:** FWER-corrected results — real (ADNI) images.

		Rejection rates					
	6 mm smoothing		12 mm smoothing		Meyer-Lindenberg et al. (2008)^a^	
	*α*_*c*_	Observed(%)	Permuted(%)^b^	Observed(%)	Permuted(%)^b^	Observed(%)	Permuted(%)^c^
*t-tests*							
Cluster size							
Stationary	0.001	10.7	9.2 ± 1.1	3.4	3.6 ± 0.7	–	–
0.01	23.3	24.8 ± 2.2	9.4	9.4 ± 1.5	–	–
0.05	47.4	46.7 ± 1.8	20.7	18.6 ± 1.5	–	–
Non-stationary	0.001	10.0	8.1 ± 1.0	3.9	3.8 ± 0.8	–	–
0.01	19.9	21.2 ± 1.6	9.1	9.3 ± 1.5	–	–
0.05	45.0	43.2 ± 2.2	20.1	18.4 ± 1.6	–	–
Voxel-wise	–	3.4	2.7 ± 0.6	3.0	2.7 ± 0.6	1.9	1.1 ± 0.4
							
*F-tests*							
Cluster size							
Stationary	0.001	13.0	10.3 ± 1.6	4.4	3.8 ± 1.1	–	–
0.01	30.9	29.6 ± 2.1	11.9	10.7 ± 1.7	–	–
0.05	60.4	60.6 ± 1.7	26.1	24.5 ± 2.5	–	–
Non-stationary	0.001	11.6	9.1 ± 1.2	4.7	4.5 ± 1.2	–	–
0.01	25.6	25.1 ± 2.1	11.4	10.9 ± 1.7	–	–
0.05	57.6	55.6 ± 2.0	25.6	23.9 ± 2.2	–	–
Voxel-wise	–	3.6	2.6 ± 0.7	3.0	2.9 ± 0.7	–	–

a Results refer to whole-brain *t*-tests using structural (VBM) data with 12 mm smoothing.

b Mean rejection rate ± SD across 10 permutations.

c Mean rejection rate ± SD for 4 permutations.

**Table 2:** FDR-corrected results—real (ADNI) images.

		Rejection rates^a^		
	*α*_*c*_	6 mm smoothing	12 mm smoothing	Meyer-Lindenberg et al. (2008)
*t-tests*				
Cluster size				
Stationary	0.001	12.7	2.7	–
0.01	31.4	8.0	–
0.05	51.6	17.7	–
Non-stationary	0.001	10.7	2.6	–
0.01	26.9	7.9	–
0.05	48.1	16.0	–
Voxel-wise	–	3.3	1.9	1.8
				
*F-tests*				
Cluster size				
Stationary	0.001	15.9	2.9	–
0.01	41.1	11.6	–
0.05	75.7	25.4	–
Non-stationary	0.001	13.6	3.3	–
0.01	35.9	11.1	–
0.05	70.3	24.4	–
Voxel-wise	–	2.9	1.6	–

a Rejection rates for unpermuted data only were considered for FDR-corrected tests.

**Table 3:** Results — simulated images.

		Rejection rates			
	6 mm smoothing		12 mm smoothing	
	*α*_*c*_	Stationary	Non-stationary^a^	Stationary	Non-stationary^b^
*t-tests*					
Cluster size					
Stationary	0.001	0.5	5.5	2.3	8.2
0.01	1.0	11.7	1.6	13.0
0.05	13.0	35.9	6.9	21.9
Non-stationary	0.001	0.6	1.1	2.7	2.6
0.01	0.9	0.3	1.4	1.9
0.05	9.3	10.7	6.4	7.4
Voxel-wise					
FWE	–	3.7	3.6	5.1	4.3
FDR	–	4.7	4.0	2.9	2.7
					
*F-tests*					
Cluster size					
Stationary	0.001	0.3	5.6	2.9	7.7
0.01	0.4	10.7	0.9	10.9
0.05	2.1	20.3	2.4	20.4
Non-stationary	0.001	0.4	0.7	3.1	2.7
0.01	0.4	0.1	0.9	2.3
0.05	1.1	1.4	2.2	2.1
Voxel-wise					
FWE	–	2.6	2.7	3.6	3.1
FDR	–	2.0	3.4	1.4	1.3

a Images constructed from concentric regions smoothed with 4, 6 and 9 mm Gaussian kernels.

b As in footnote a with 8, 12 and 18 mm Gaussian kernels.

## Footnotes

2 Anticonservative tests produce *P*-values that are too small, giving rise to rejection (false positive) rates that are higher than the nominal (expected) rate for the test.

3 Precisely the roughness measure is pooled and then converted to FWHM in order to minimise bias; see (Worsley et al., 1999) for details.
